# An Intradermal Inoculation Mouse Model for Immunological Investigations of Acute Scrub Typhus and Persistent Infection

**DOI:** 10.1371/journal.pntd.0004884

**Published:** 2016-08-01

**Authors:** Lynn Soong, Nicole L. Mendell, Juan P. Olano, Dedeke Rockx-Brouwer, Guang Xu, Yenny Goez-Rivillas, Claire Drom, Thomas R. Shelite, Gustavo Valbuena, David H. Walker, Donald H. Bouyer

**Affiliations:** 1 Department of Pathology, Center for Biodefense and Emerging Infectious Diseases, Center for Tropical Diseases, Sealy Center for Vaccine Development, Institute of Human Infections and Immunity, School of Medicine, University of Texas Medical Branch, Galveston, Texas, United States of America; 2 Department of Microbiology and Immunology, University of Texas Medical Branch, Galveston, Texas, United States of America; University of Liverpool, UNITED KINGDOM

## Abstract

Scrub typhus is a neglected tropical disease, caused by *Orientia tsutsugamushi*, a Gram-negative bacterium that is transmitted to mammalian hosts during feeding by *Leptotrombidium* mites and replicates predominantly within endothelial cells. Most studies of scrub typhus in animal models have utilized either intraperitoneal or intravenous inoculation; however, there is limited information on infection by the natural route in murine model skin or its related early host responses. Here, we developed an intradermal (i.d.) inoculation model of scrub typhus and focused on the kinetics of the host responses in the blood and major infected organs. Following ear inoculation with 6 x 10^4^ *O*. *tsutsugamushi*, mice developed fever at 11–12 days post-infection (dpi), followed by marked hypothermia and body weight loss at 14–19 dpi. Bacteria in blood and tissues and histopathological changes were detected around 9 dpi and peaked around 14 dpi. Serum cytokine analyses revealed a mixed Th1/Th2 response, with marked elevations of MCP-1/CCL2, MIP-1α/CCL3 and IL-10 at 9 dpi, followed by increased concentrations of pro-inflammatory markers (IL-6, IL-12, IFN-γ, G-CSF, RANTES/CCL5, KC/CCL11, IL-1α/β, IL-2, TNF-α, GM-CSF), as well as modulatory cytokines (IL-9, IL-13). Cytokine levels in lungs had similar elevation patterns, except for a marked reduction of IL-9. The *Orientia* 47-kDa gene and infectious bacteria were detected in several organs for up to 84 dpi, indicating persistent infection. This is the first comprehensive report of acute scrub typhus and persistent infection in i.d.-inoculated C57BL/6 mice. This is a significant improvement over current murine models for *Orientia* infection and will permit detailed studies of host immune responses and infection control interventions.

## Author Summary

Scrub typhus is a life-threatening disease that presents as a severe acute febrile illness. It is caused by mite-transmitted *Orientia tsutsugamushi*, a Gram-negative, obligately intracellular bacterium. Every year, approximately one million people are infected globally; however, there is no vaccine for the control of this infection. Mechanistic studies of host immune responses have been few, partially due to the limited availability of suitable animal models and research facilities. Here, we report our development of an intradermal inoculation mouse model that mimics the natural infection. We also examined the kinetics of immune and inflammatory responses during acute and chronic stages of *O*. *tsutsugamushi* infection. As the first comprehensive report of an intradermal inoculation murine model of scrub typhus, this study improves our understanding of host immune responses following cutaneous exposure to the bacteria and opens new avenues for future vaccine-based investigations.

## Introduction

Scrub typhus (Tsutsugamushi disease) is a potentially severe acute febrile illness transmitted through the bite of an infected feeding larval mite or chigger [1]. It is caused by *Orientia tsutsugamushi* (formerly known as *Rickettsia tsutsugamushi* before 1995 when it was reassigned to the new genus *Orientia*), a strictly intracellular, Gram-negative bacterium that resides free in the cytoplasm of the microvascular endothelium [2]. Phylogenetically, *Orientia* is in the order Rickettsiales, family Rickettsiaceae [3,4]. The vector and reservoir in nature of *O*. *tsutsugamushi* are *Leptotrombidium spp*. mites or chiggers [5,6,7]. However, these parasites in both nymphal and adult stages do not feed on vertebrates and are therefore irrelevant in transmission of human disease. Scrub typhus is prevalent in a large geographic area, comprising approximately 13,000,000 km^2^, stretching from the Russian Far East, China, Japan and Korea in the north, to northern Australia in the south, and to Afghanistan and Pakistan to the west [3,8]. Many islands in the Pacific Ocean and Indian Ocean, including Taiwan, The Philippines, Indonesia and Sri Lanka among others, also have reported cases. Accordingly, more than 1 billion people living in those areas are at risk of acquiring the infection, and overall more than 1 million cases are estimated to occur every year [9]. Historically, this disease had a dramatic impact on U.S. troops during World War II and the Vietnam War and is now emerging as an important disease in the Far East [3,10,11]. Scrub typhus is rated very high on the Armed Forces Medical Intelligence Center’s Global Severity Risk Index assessment of risk of naturally acquired infections of U.S. military personnel [12]. In 1999, the World Health Organization referred to scrub typhus as “probably one of the most underdiagnosed and underreported febrile illnesses requiring hospitalization in the region.”

Scrub typhus is responsible for a large proportion of severe undifferentiated fevers, as well as up to 23% of all febrile episodes in rural endemic areas and has relatively high mortality rates [13]. The average case-fatality rate is ~10%, but has been reported to be as high as 35% in some series, mostly due to delays in initiating effective antimicrobial treatment. In the pre-antibiotic era, case-fatality ratios could be as high as 50% [14]. However, the disease spectrum is extremely broad, and it is likely that disease severity in humans might depend, in part, on the strain of *O*. *tsutsugamushi* involved in human cases. In fact, more than 70 strains of *O*. *tsutsugamushi* are currently described [15]. Reinfection with the same genotype is possible in highly endemic areas. Some individuals can progress to persistent infection even after antibiotic treatment [16], and there are no effective vaccines for scrub typhus [4,17]. Adaptive immunity or cross-protection following *O*. *tsutsugamushi* infection in humans appears strain-related and short-lived [13,18,19,20], but the underlying mechanisms of waning immunity are largely unclear.

Non-human primates, especially *Macaca fascicularis* (cynomolgus macaques) and *Presbytis cristatus* (silvered leaf monkeys), have been used to study the histopathology and immunological responses to *O*. *tsutsugamushi* [21,22,23,24]. Walsh and colleagues have used cynomolgus macaques to evaluate the clinical manifestations and antibody responses, as well as histological features of eschars, a unique and localized pathological skin lesion often occurring in humans following inoculation of the organism at a cutaneous mite feeding site [22]. More recently, Paris and colleagues have developed a cynomolgus macaque intradermal (i.d.) challenge model that closely mimics natural infection and eschars in humans and have used it to evaluate protective immune responses induced by p47-DNA vaccination against infection with *O*. *tsutsugamushi* Karp strain (OtK) [25]. They have provided the first phenotypic correlations of immune protection in scrub typhus. While non-human primates are the best models for human scrub typhus, they are not widely used in laboratories due to the high expense and other logistical issues. Thus, there is a great need to develop murine models that mimic the natural entry route of organism inoculation and manifest certain immunological and pathogenic features of human scrub typhus.

Elucidation of pathogenic mechanisms or protective immunity to OtK has been hampered by the lack of availability of well-standardized rodent animal models that mimic the pathological features of the human disease. Most publications report murine models that were initiated via the intraperitoneal (i.p.) inoculation route in outbred mouse strains, which resulted in diffuse peritonitis and severe mesothelial infection of the peritoneum, rather than disseminated, systemic infections following an incubation period [26,27,28,29,30]. The newly developed model of intravenous (i.v.) inoculation in C57BL/6 mice leads to disseminated infection of endothelial cells, and lesions resembling the human pathology in cases of fatal scrub typhus [31,32]. However, this model bypasses the natural route of infection since infections transmitted in nature follow i.d. entry of the organisms. Several groups have explored other routes of infection via subcutaneous (s.c.) or i.d. needle inoculation or mite-based transmission; however, most of these reports have focused on the early phases of infection in BALB/c mice [33], or in outbred mice which cannot be utilized for reproducible mechanistic studies [34,35,36].

Here, we report a sub-lethal murine model for acute scrub typhus and persistent infection following i.d. inoculation of C57BL/6 mice with OtK. This represents an advance that complements our recent development of a lethal scrub typhus model that used i.v. inoculation [31,32]. Validation of this i.d. inoculation model should permit in-depth mechanistic studies related to the pathogenesis and specific immunological investigations of the host immune response following route-specific exposure to the bacteria, and it will open new avenues for future vaccine- or immune-based investigations for disease control.

## Materials and Methods

### Mouse infection and ethics statement

Female wild-type C57BL/6 (B6, from Harlan Laboratories, Indianapolis, IN) were used in this study. Mice were maintained under specific pathogen-free conditions and used at 8–10 weeks of age, following protocols (#9007082B and #1302003) approved by the Institutional Animal Care and Use Committee at the University of Texas Medical Branch (UTMB) in Galveston, TX. All mouse infection studies were performed in the ABSL3 facility in the Galveston National Laboratory located at UTMB; tissue processing and analysis procedures were performed in the BSL3 or BSL2 facilities. All procedures were approved by the Institutional Biosafety Committee, in accordance with Guidelines for Biosafety in Microbiological and Biomedical Laboratories. UTMB operates to comply with the USDA Animal Welfare Act (Public Law 89–544), the Health Research Extension Act of 1985 (Public Law 99–158), the Public Health Service Policy on Humane Care and Use of Laboratory Animals, and the NAS Guide for the Care and Use of Laboratory Animals (ISBN-13). UTMB is a registered Research Facility under the Animal Welfare Act, and has a current assurance on file with the Office of Laboratory Animal Welfare, in compliance with NIH Policy.

### Bacterial cultures and stock propagation

The identity of OtK bacterium was confirmed by sequencing of the *Orientia* 47-kDa gene (accession #L31934), prior to growth in Vero cells; oriential stock was prepared from heavily (80–100%) infected Vero cell cultures, as previously described [37]. To produce high-titer bacterial stocks and to avoid loss of virulence through laboratory passage adaptation, we prepared mouse lung-derived bacterial stocks by passages through B6 mice, as previously reported [31]. Briefly, B6 mice were inoculated i.v. with Vero cell-propagated OtK and were euthanized when they were at the peak of illness. Lung tissues were homogenized; bacteria were isolated, aliquoted for titer/quality analyses, stored at -80°C in sucrose-phosphate-glutamate (SPG, 218 mM sucrose, 3.76 mM potassium phosphate monobasic, 7.1 mM potassium phosphate dibasic, 4.9 mM potassium glutamate) buffer, or utilized to inoculate a naïve group of animals for further amplification. The animal passages were performed 3–5 times to create high-quality bacterial stocks; the same lot of stock was used for all experiments described in this study.

### Viability assay and bacterial load determination

Confluent monolayers of Vero cells in 6-well plates were inoculated with 200 μl of OtK stocks (in serial 10-fold dilutions in triplicate). After 2 h of incubation at 34°C with 5% CO_2_, plates were triple rinsed with warm phosphate-buffered saline (PBS) to remove bacteria which did not adhere or invade the monolayer, and DNA was extracted for bacterial load analysis via quantitative PCR (qPCR), as in our reports [31,32]. Briefly, the 47-kDa protein gene was amplified via specific primers OtsuF630 (5′-AACTGATTTTATTCAAACTAATGCTGCT-3′) and OtsuR747 (5′-TATGCCTGAGTAAGATACGTGAATGGAATT-3′) (IDT, Coralville, IA). Serial 10-fold dilutions of known concentrations of single 47-kDa gene-containing plasmid were utilized to determine the copy number. DNA isolated from bead-homogenized tissue samples were used to assess tissue bacterial loads. Sample were normalized with the mouse GAPDH gene (F, 5′-CAACTACATGGTCTACATGTTC-3′; R, 5′-CTCGCTCCTGGAAGATG-3′, IDT). Data are presented as 47-kDa copy numbers per 10^5^ or 10^6^ copies of GAPDH for tissues, or per μl of blood.

### Inoculation of animals

B6 mice were purchased at 7–9 weeks of age and allowed to acclimatize for 7 days prior to experimental use. To select the experimental dose, mice were inoculated with OtK or PBS (a sham control) in the dermis of the lateral ear with a range of doses [6 x 10^5^ to 6 x 10^1^ viable organisms in 10–12 μl delivered via a 30G-needle and 25-μl Hamilton syringe (Hamilton Company, Reno, NV)]. Mice were monitored twice daily for 28 days for signs of illness. Clinical signs of illness were consistently observed between 10–13 dpi with 10^3^ to 10^5^ viable organisms, whereas animals that received lower doses (<10^3^ total organisms) had delayed, sporadic onset of clinical signs of illness that occurred between 14–16 dpi. A dose of 6x10^4^ was selected for subsequent experiments for the purposes of consistency of inoculation concentration and consideration that the dose of natural transmission of *O*. *tsutsugamushi*, although currently unknown, would not likely be as great as 10^5^ or higher organisms. Mice were inoculated as described with 6x10^4^ viable organisms or PBS and monitored for signs of illness daily (ruffled fur, lethargy, erythema, temperature change, and weight loss) as in our previous studies [31,32]. Body temperature and weight were monitored daily from 0–21 dpi, and then weekly during the remaining period of study. During the 1^st^ week of infection, a group of mice (n = 4) was sacrificed daily for assessment of bacterial loads in blood and organs and for histology. During the 2^nd^ week of infection, mice were sacrificed every other day until 21 dpi and then once weekly until 84 dpi.

### Determination of viability of persistent bacteria

To determine if bacterial DNA detected in tissues represented viable organisms, we collected tissues at 81–84 dpi from 4 mice (with IgG titers of 1:65,536) in the first experiment, and from 3 mice (with IgG titers of 1:32,768) in the second. Lung, kidney, and spleen/liver/lymph node samples were placed in DMEM, homogenized by using a 7-ml glass Dounce homogenizer in cold SPG buffer, and centrifuged at 700 x g for 10 min at 4°C. Supernatants were saved, while pellets were subjected to another round of homogenization and centrifugation. Bacteria in supernatants were harvested by centrifugation at 22,000 x g for 45 min at 4°C. Enriched bacterial pellets were re-suspended and pooled in a total of 10 ml of SPG. Each naïve mouse was inoculated via the i.p. route with a 250-μl aliquot (3–4 mice per tissue group). Mice were monitored daily for signs of illness for 21 days.

### Hematologic analysis

At the desired time points, blood samples (500 μl) were collected from each mouse into K_2_EDTA-coated microtainer tubes (Becton Dickinson, Franklin Lakes, NJ). Blood cell counts were measured by using a calibrated 950FS HemaVet apparatus (Drew Scientific, Waterbury, CT). Blood samples were analyzed by using the FS-Pak reagent kit, for measuring white blood cell count, differential leukocyte (%) count, hemoglobin, hematocrit, red blood cell count, red cell distribution width, platelet count, and mean platelet volume, respectively.

### Indirect immunofluorescence assay

Antigen-coated, acetone-permeabilized 12-well slides were equilibrated to room temperature in PBS and then blocked in PBS containing 1% bovine serum albumin (BSA) and 0.01% sodium azide for 10 min. Sera were diluted 2-fold starting at 1:64 and, if reactive, serially diluted to final end point titers in a solution of PBS containing 1% BSA, 0.1% Tween 20, and 0.01% sodium azide. Dilutions of sera were added to antigen-coated wells and incubated at 37°C for 30 min in a humidified chamber. Slides were rinsed twice with PBS containing 0.1% Tween-20 for 10 min. A secondary antibody, DyLight 488-conjugated anti-mouse IgG (1:15,000, Jackson ImmunoResearch, West Grove, PA) was added and incubated for 30 min at 37°C in a humidified chamber. Slides were washed twice as before, with the final wash containing 1% Evans blue solution, mounted with DAPI fluoromount-G (SouthernBiotech, Birmingham, AL) and coverslipped. Slides were observed under a fluorescence microscope at 400X magnification (Leica Microsystems, Buffalo Grove, IL).

### Serum cytokine measurement

Blood was collected from mice at the time of euthanasia via cardiac puncture and sera were stored at -20°C until use. Serum samples (100 μl) were used for cytokine measurement in a Mouse Cytokine 23-Plex assay (Bio-Rad Laboratories, Hercules, CA), according to the manufacturer's instructions. Samples with cytokine concentrations below the detection limits were assigned an averaged value between 0 and the lowest detectable levels in each assay; all samples were retained in the data set.

### Histopathology and immunohistochemical (IHC) staining

All tissues were fixed in 10% neutral-buffered formalin and embedded in paraffin, and sections (5-μm thickness) were stained with hematoxylin and eosin or processed for antibody (Ab) staining, as in our previous reports [32]. For IHC staining, all reagents were from Vector Laboratories (Burlingame, CA), unless specified. Briefly, slides were sequentially processed for antigen retrieval, deparaffinization, and rehydration. Sections were blocked with 1X casein (for endogenous IgG), a BLOXALL blocking solution (for endogenous alkaline phosphatase), an avidin and biotin blocking solution, and normal goat serum (for non-specific binding sites). Sections were incubated with rabbit anti-OtK Ab (1:12,000, produced in our laboratory) at 4°C overnight, followed by incubation with biotinylated goat anti-rabbit IgG (1:200) for 30 min. Signals were detected by incubation with alkaline phosphatase conjugate (1:200) and developed with an alkaline phosphatase substrate kit. Slides were counterstained with hematoxylin, dehydrated, mounted with VectaMount, and examined under an Olympus BX53 microscope.

### Analysis of histopathology in the lung, liver and spleen

All slides were examined and scored blindly by two pathologists (without knowledge of dpi or bacterial loads), following the below criteria. For hepatic pathology scores, the diameters of clusters of inflammatory infiltrates were measured, and the average lesion size and number of lesions per 10 medium-power fields (100X) were determined for each time point. A liver inflammatory index was calculated as: number of lesions per 10 medium-power fields multiplied by the mean diameter of mononuclear infiltrative clusters (μm). For pulmonary pathology scores, all lung sections were examined sequentially (according to dpi) to obtain a general assessment of the histopathology and establish grading parameters; grades 1–4 were scored based on lesion spectra throughout the entire course of infection. Grade 1+: widening of alveolar septa with scattered inflammatory cells in focal areas of pulmonary parenchyma, and focal inflammatory cells around bronchovascular bundles. Grade 2+: grade 1 criteria plus multifocal clusters of inflammatory infiltrates around bronchovascular bundles. Grade 3+: widening of alveolar septa with diffuse inflammatory cell infiltrates present in the pulmonary parenchyma, bronchovascular bundles, and focal areas of atelectasis. Grade 4+: grade 3 criteria plus extensive areas of atelectasis. Splenic histopathology was assessed based on changes in the white pulp, specifically expansion of the marginal zone and lymphoid activation in periarteriolar lymphoid sheaths.

### Statistical analysis

Data were presented as mean ± standard errors of the mean (SEM). Statistical significance of differences between individual treatment and control groups was determined by using Student’s t test. One-way ANOVA and Tukey’s post-test were used for multiple group comparisons. Statistically significant values are designated as *, *p* < 0.05; **, *p* < 0.01; ***, *p* < 0.001, respectively.

## Results

### Clinical manifestations during acute and chronic stages of the infection

**Fig 1:**
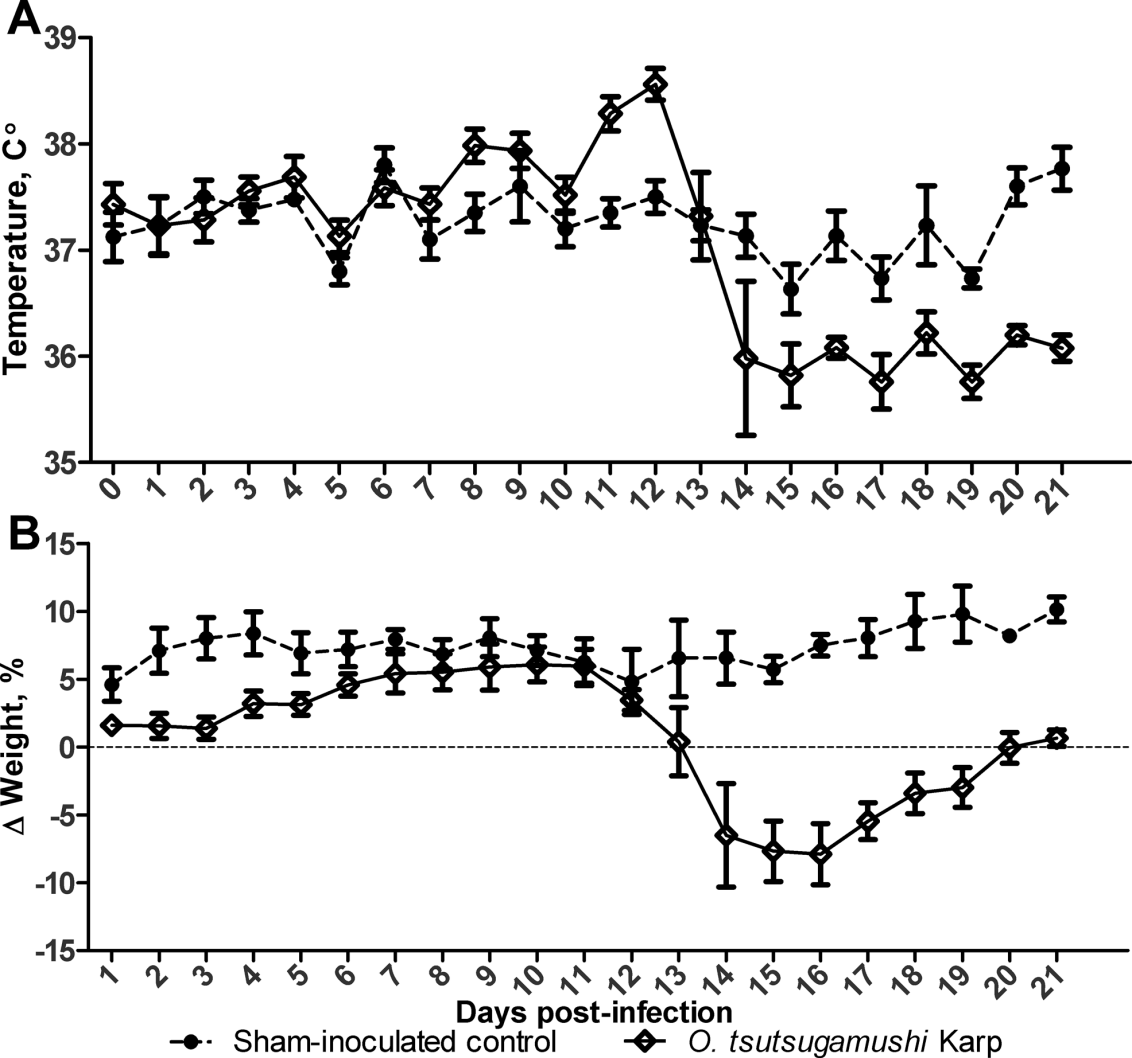
Pathophysiologic responses following infection with *O*. *tsutsugamushi* Karp strain. **A**) A mean increase in body temperature was observed from 11–12 dpi, followed by hypothermia at 14 dpi. **B**) Weight loss was presented as the percentage of body weight change compared with 0 dpi. Weight loss was evident from 13–16 dpi, followed by a gradual recovery beginning around 17 dpi.

Following i.d. inoculation in the ear, we observed no eschar formation at the inoculation site and no clinical signs of illness during the first 7 dpi, a finding similar to those in reported studies for i.d. inoculation in Swiss CD-1 outbred mice [35] and s.c. inoculation in BALB/c mice [33]. Infected B6 mice had elevated body temperatures at 11 and 12 dpi (38.2–38.7°C), followed by hypothermia from 14 to 21 dpi (**Fig 1A**), and recovered to normal body temperature by 28 dpi. Infected mice showed an up to 13% difference in weight change compared to that in sham-inoculated controls (**Fig 1B**). Weight loss was most evident at 14–16 dpi, followed by gradual recovery starting at 17 dpi. By 20 dpi, the body weight of infected mice had reached the pre-infection level, but it remained much lower than that of sham-inoculated controls. To assess infection outcomes, we examined circulating OtK-specific IgG titers by IFA, as well as blood and tissue bacterial loads via PCR-based quantification of the OtK 47-kDa gene, from 1 until 84 dpi. Seroconversion was observed as early as 5 dpi (1:128 in 25% of mice), reached the peak reciprocal endpoint titer of 1:65,536 by 13 dpi, and sustained high-titers (1:65,536) even at 84 dpi (or 12 weeks) (**S1 Fig**). Our findings were consistent with, but extended from, previous reports in human cases [38,39].

**Fig 2:**
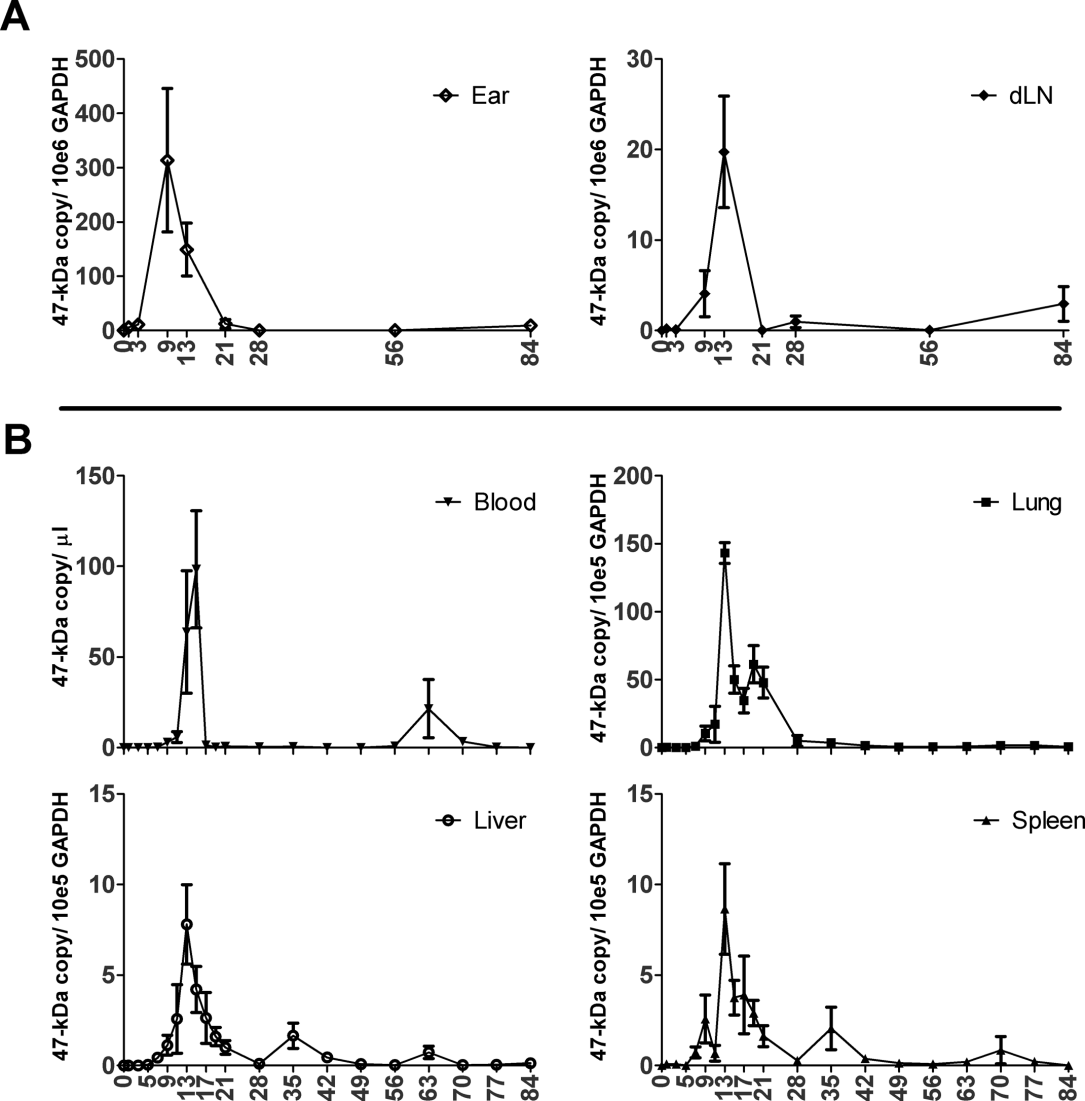
Bacterial loads following infection with *O*. *tsutsugamushi* Karp strain (OtK). **A**) Quantification of the OtK 47-kDa gene in the ear, draining lymph nodes (dLN), or **B**) in blood, lung, liver, spleen, and brain tissues. All data are presented as 47-kDa gene copies per 10^5^ or 10^6^ GAPDH for tissues, or per μl of blood. Representative data from one of two independent experiments are shown.

Daily analyses of blood and tissue bacterial loads during the first 28 days of infection, as well as weekly analyses until 84 dpi, revealed the following consistent features. The earliest peak of bacterial burden occurred in the ear, the site of inoculation, at day 9 pi. At 1–7 dpi, bacterial loads were low or undetectable in the blood, lungs, liver, spleen (**Fig 2**), and brain (**S2 Fig)**. In blood, bacteria were consistently detected at 9 dpi, followed by a peak mean bacteremia at 15 dpi (98.4 copies/μl blood). In the lungs, bacterial loads peaked at 13 dpi (143.2 copies/10^5^ GAPDH copies) and remained detectable at 35 dpi. The liver, spleen, and brain bacterial loads were lower, but were sustained longer. In the liver, bacterial loads peaked at 13 dpi (7.8 copies/10^5^ GAPDH copies) and remained detectable at 63 dpi. In the spleen and brain, bacterial loads peaked at 13 dpi and remained detectable at 70 dpi. To confirm earlier reports of persistent *Orientia* infection in humans and animal models [16,40,41], we selected the kidney as an additional organ to analyze during late infection. Bacteria were observed in the kidney from 70–77 dpi. The peaks of bacterial loads around 13–15 dpi corresponded with the time of occurrence of the greatest reduction of body weight in the infected mice.

The prolonged presence of OtK DNA in the ear, dLN, blood, liver, spleen, and brain (63–84 dpi), as well as in the kidneys (70–84 dpi, **S2 Fig**), led us to investigate whether viable infectious organisms were present. At 81–84 dpi, we prepared homogenates from the kidney, lung, and spleen/liver/lymph nodes and used them respectively to inoculate naïve B6 mice via the i.p. route (3–4 mice per group), the most sensitive method to detect low quantities of *Orientia* [31]. Our data from two independent experiments revealed that mice that were inoculated i.p. with kidney homogenates had the highest mortality rates and tissue bacterial loads; 57.1% (4 out of 7) mice died between 12–15 dpi, with 2x10^4^, 3.81x10^6^, 4.54x10^6^, and 1.25x10^7^ *Orientia* 47-kDa copies per mg of kidney of the inoculated mice, respectively. The remaining 3 mice inoculated with kidney homogenates all showed clinical signs of illness on 11 to 15 dpi, but recovered. Mice inoculated with lung homogenates had a lower mortality rate and tissue bacterial loads, as 1 out of 3 tested mice (33%) died at 14 dpi, with 7.07x10^5^ *Orientia* 47-kDa copies per mg of lung of the inoculated mice. Mice inoculated with spleen/liver/lymph node homogenates recovered from mild illness and were sacrificed around 21 dpi. Together, we concluded that even though i.d.-inoculated mice had high titers of OtK-specific IgG (1:32,768 or 1:65,536), they had infectious bacteria that persisted in the kidneys and other tissues, and, accordingly, these mice represent a model of observations of persistent infection in human scrub typhus [16].

### Hematological and histopathological analyses

To better understand the clinical features of this i.d. model, we performed hematological and histopathological analyses. While white blood cell counts remained relatively normal between 1–13 dpi, infected mice had transient, but consistent, alterations in the platelet and red blood cell counts and cell morphology. The thrombocytopenia was transient, but most severe at 13 dpi during the peak of endothelial infection, likely reflecting deposition of platelets in foci of endothelial injury (**S3 Fig**). The transient increases in mean platelet volume (at 13 dpi) corresponded to the period of the bone marrow response for the release of immature platelets. Similarly, the increased red cell distribution width on 21 dpi, and from then to the end of the study, may reflect the release of newly formed erythrocytes from the bone marrow in response to the development of anemia on 9 dpi.

**Fig 3:**
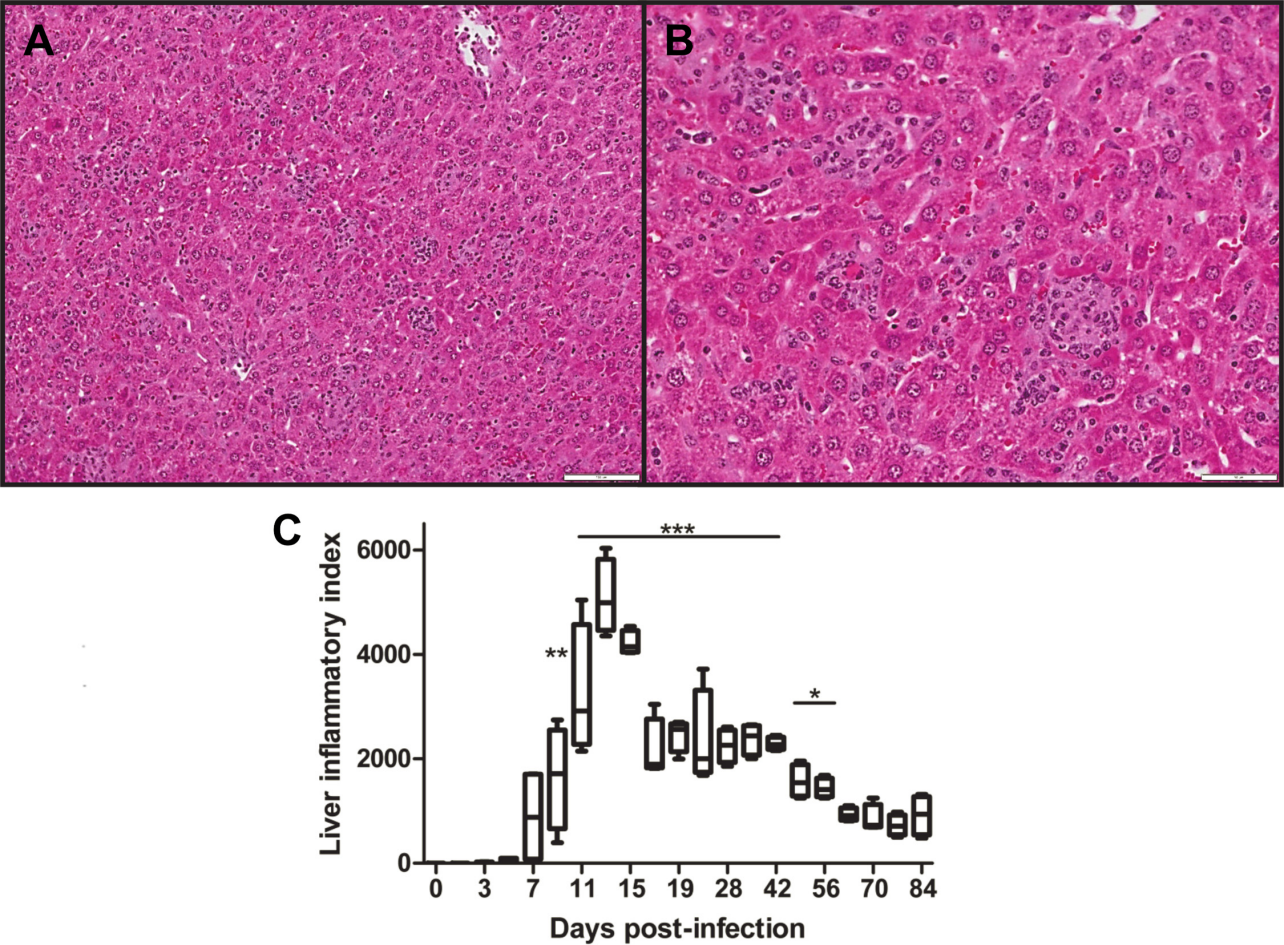
Histopathologic changes in mouse liver after infection with *O*. *tsutsugamushi*. **A**) Liver sections collected at 9 dpi, showed scattered intraparenchymal clusters of mononuclear inflammatory cells located randomly in the hepatic lobules (100X). **B**) Medium magnification shows lymphohistiocytic clusters between hepatic cords, (200X). **C**) Liver inflammatory index (inflammatory cluster per 10 medium-power fields x diameter of cluster, μm). Scores of infected groups were compared with sham controls, and statistically significant differences were observed from 9–56 dpi, respectively. *, *p* < 0.05, **, *p* < 0.01, *** *p* < 0.001.

Examination of the liver in infected animals showed foci of mononuclear inflammatory cells at 7 dpi, and a higher inflammatory index was statistically significant starting on 9 dpi (*p* < 0.01, **Fig 3**). The diameter and number of lesions in the liver peaked between 11 and 13 dpi (*p* < 0.01), which coincided with the highest bacterial loads in the liver (Fig 2). After day 13, the number of lesions in the liver decreased and then plateaued, but the inflammatory index remained elevated until 56 dpi, during which time the pathologic foci increased in size and then evolved from discrete clusters to confluent patches of mononuclear cellular infiltrates as the disease progressed. Lesions in the spleen progressed similarly, as characterized by marked expansion of the marginal zone and lymphoid expansion in peri-arteriolar lymphoid sheaths, which were present even at 84 dpi. Representative images of the kidney and spleen revealed persistent multifocal interstitial inflammatory infiltrates (**S4 Fig**). Examination of brain sections during the infection revealed no overt pathological lesions.

**Fig 4:**
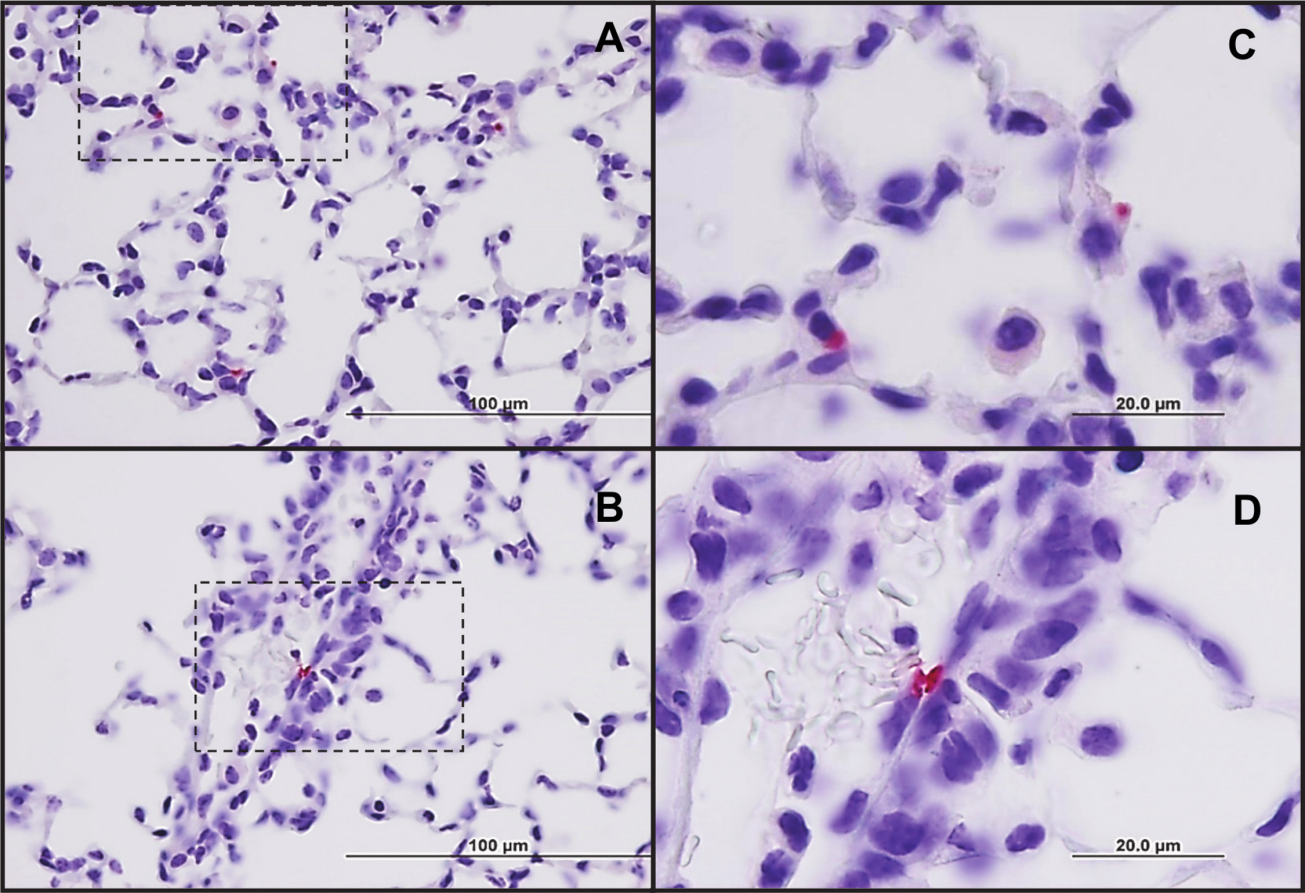
Location of *O*. *tsutsugamushi* Karp strain antigens in the lungs at 12 days following i.d. inoculation. Low-power sections of the lungs revealed the presence of *Orientia* antigens (red) in alveolar septa (**A**) and an interstitial capillary (**B**) (100X, bars = 100 μm). High power magnification (400X) revealed the presence of *Orientia* in septal (**C**) and interstitial capillaries (**D**); bars = 20 μm.

**Fig 5:**
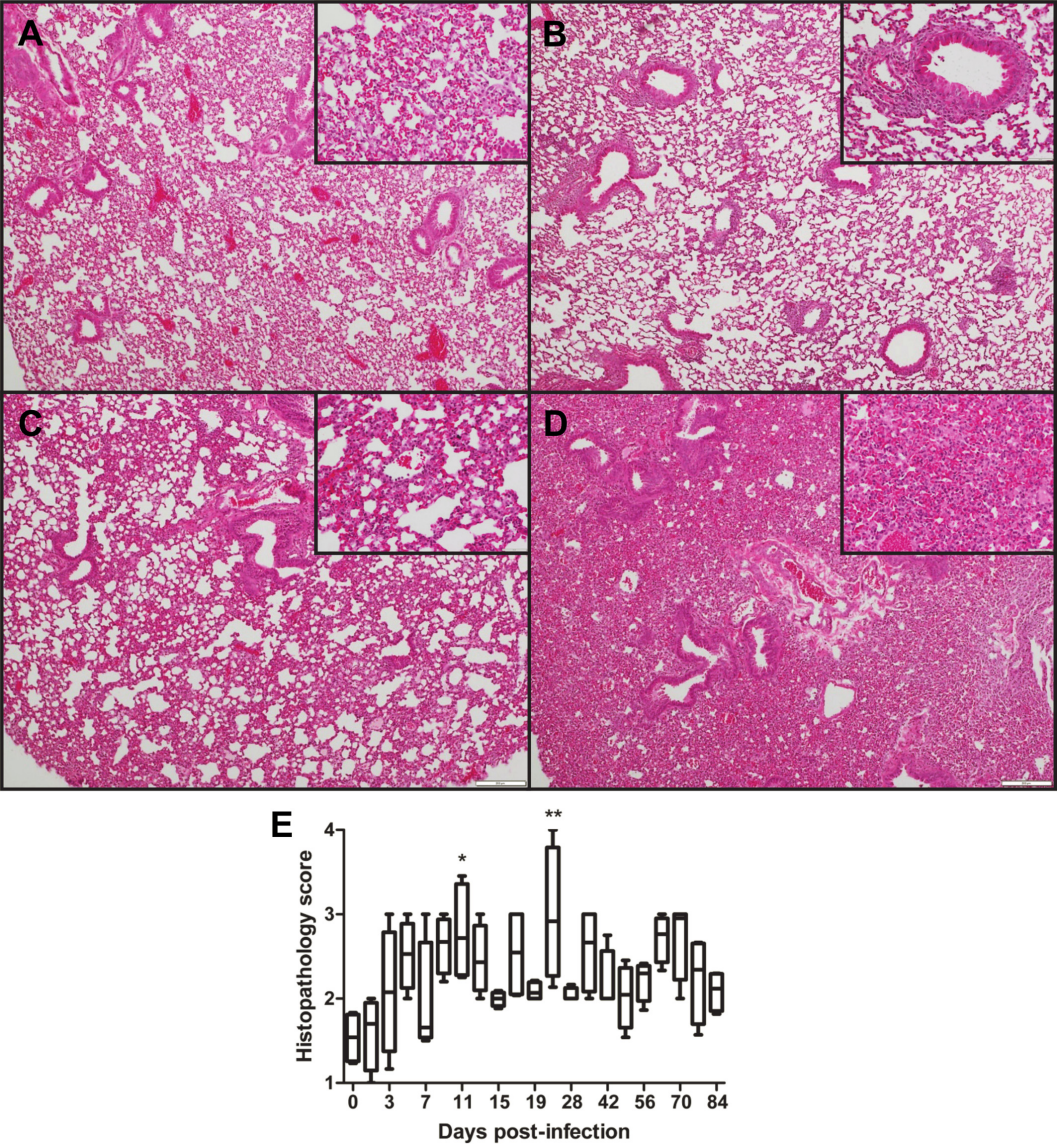
Histopathologic changes in mouse lung after infection with *O*. *tsutsugamushi*. Lung tissues collected between 9 and 11 dpi were stained with H&E and scored from grades 1 to 4. **A**) Grade 1: widening of alveolar septa and scattered inflammatory cells. **B**) Grade 2: Diffuse widening of alveolar septal walls and mononuclear inflammatory cells around bronchovascular bundles (inset). **C**) Grade 3: Marked diffuse interstitial pneumonitis with mononuclear inflammatory cells and focal areas of alveolar collapse. **D**) Grade 4: Marked diffuse interstitial pneumonitis with mononuclear inflammatory cells and large areas of alveolar collapse. All images were taken at 40X, and insets were taken at 200X magnification. **E**) Histopathology scores of infected lungs were compared with uninfected controls and presented as Whiskers 5–95% percentile. *, *p* < 0.05, **, *p* < 0.01.

Lungs are the most important and severely affected organ in OtK infection following i.v. inoculation [31,32]. Because i.d.-inoculated mice had the highest bacterial loads in the lungs, other than the inoculation site (**Fig 2**), we examined the location of oriential antigens in the lungs by OtK-specific IHC. At 12 dpi, the lungs contained oriential antigens in endothelial cells lining septal capillaries (**Fig 4**). Analysis of lung histopathology revealed substantial inter-animal variations in pathology scores during the course of infection, with most animals scoring between grades 2 and 3. The most severe pathology (grade 4) occurred at the late stage of disease (at 21 dpi) (**Fig 5A–5D**). Histological lesions in the lungs did not show resolution even at 84 dpi (**Fig 5E**).

### Serum and lung cytokine and chemokine production during acute infection

**Fig 6:**
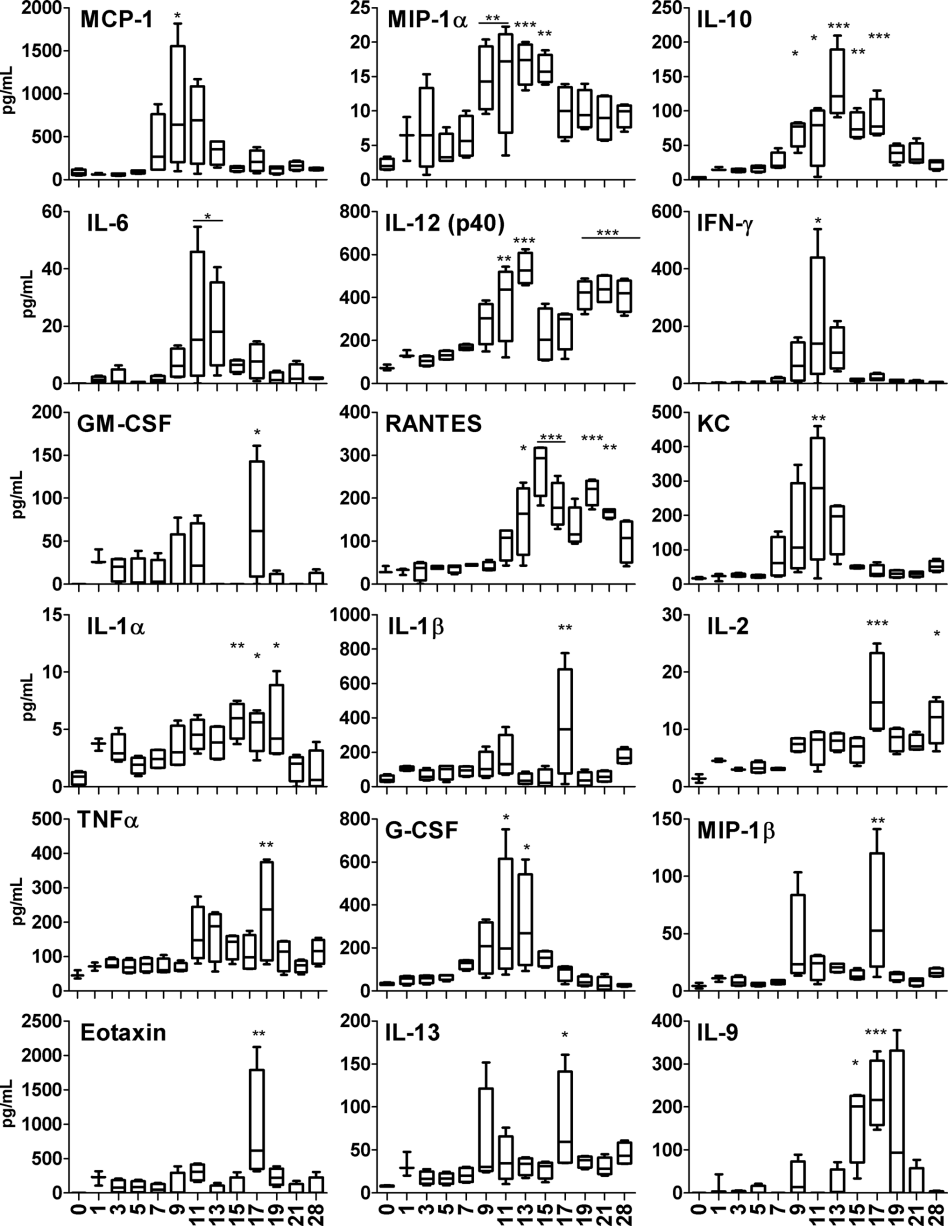
Serum cytokine levels during infection with *O*. *tsutsugamushi*. Serum samples were collected from sham controls (n = 3) and infected mice (n = 4) for measuring the indicated cytokines by using a BioPlex assay. Data (pg/ml) are presented as Whiskers 5–95% percentile. Infected samples were compared with sham controls. *, *p* < 0.05, **, *p* < 0.01, *** *p* < 0.001.

We have previously reported that following i.v. inoculation of a lethal dose of OtK in B6 mice, the development of strong type 1 immune responses, with no IL-4/IL-13 production, contributes to mouse mortality [32]. To define the immune responses in i.d.-inoculated mice, we measured cytokine profiles in the sera and lung homogenates by a BioPlex assay. Serum cytokine analyses revealed two distinct patterns (**Fig 6**). The early (9 dpi) production of a set of cytokines and chemokines (MCP-1/CCL2, MIP-1α/CCL3, and IL-10) during the incubation period was followed by the production of pro-inflammatory markers (IL-6, IL-12p40, IFN-γ, G-CSF, RANTES/CCL5, and KC/CXCL11) at 11–13 dpi, correlating with the onset of fever. The production of other cytokines (IL-1α/β, IL-2, TNF-α, GM-CSF, MIP-1β/CCL4, eotaxin/CCL11, IL-9, and IL-13) around 15–19 dpi, correlated with disease progression. At 21–28 dpi, serum markers had returned to basal levels, except for IL-12p40 and RANTES/CCL5. At several time points between 35–77 dpi, relatively low, but statistically significant, elevations of MIP-1α/CCL3, IL-1α/β, TNF-α, IL-2, IL-12p40, and IL-13 were detected, implying sustained immune responses.

**Fig 7:**
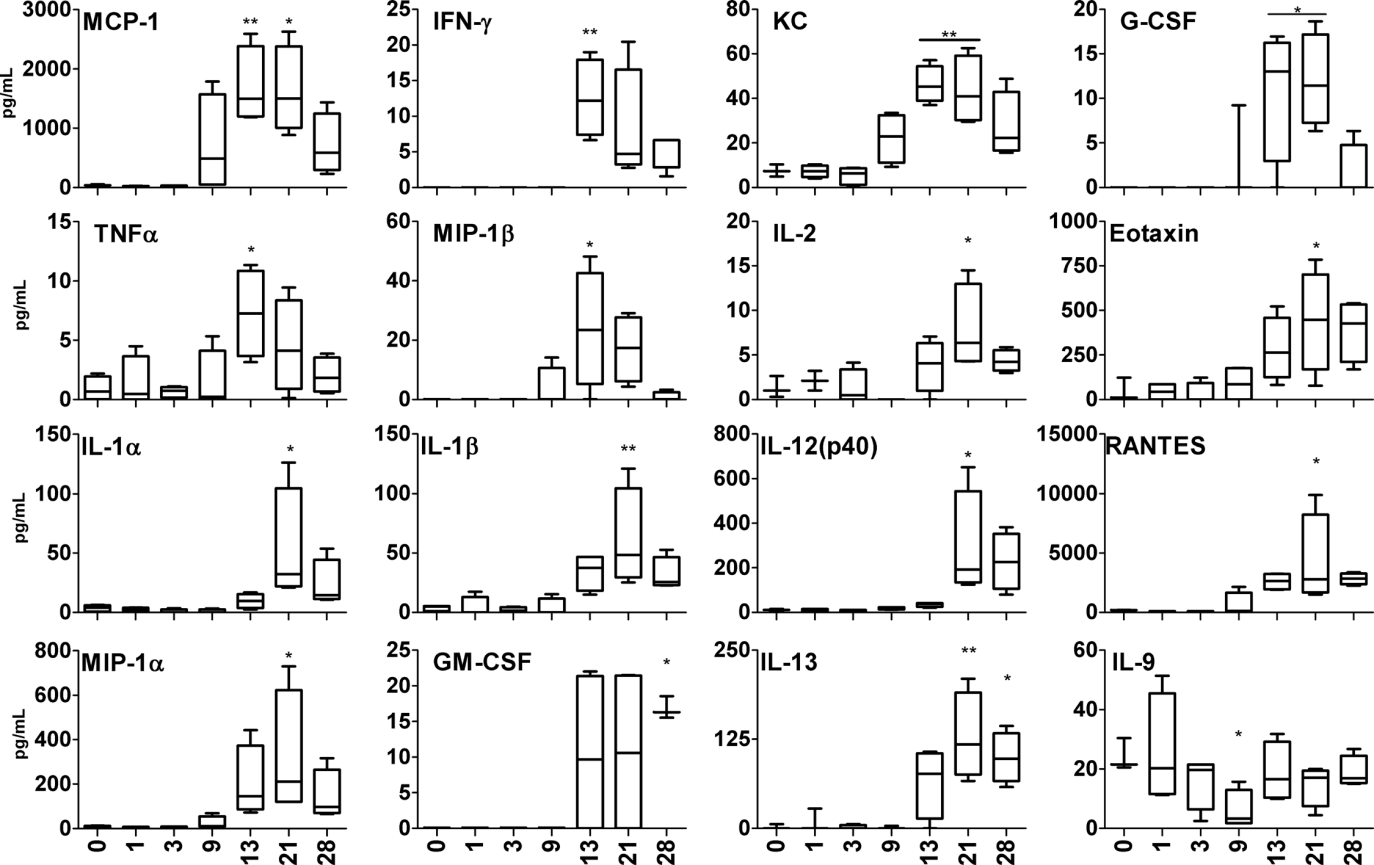
Lung cytokine levels during infection with *O*. *tsutsugamushi*. Lung samples were collected from sham controls (n = 3) and infected mice (n = 4) for measuring the indicated cytokines by using a BioPlex assay. Data (pg/ml) are presented as Whiskers 5–95% percentile. Infected samples were compared with sham controls. *, *p* < 0.05, **, *p* < 0.01.

Lung homogenates had similar cytokine and chemokine profiles as the sera (**Fig 7**). The transient elevations of MCP-1/CCL2, MIP-1β/CCL4, IFN-γ, TNF-α, G-CSF, and KC/CXCL11 at 13 and/or 21 dpi were followed by increased concentrations of IL-1α/β, IL-2, IL-12p40, RANTES/CCL5, MIP-1α/CCL3, IL-13, and GM-CSF at 21 and/or 28 dpi. Of note, IL-9 levels in lung samples were significantly reduced compared with those from sham controls, which was in sharp contrast to the IL-9 production pattern in sera.

## Discussion

Scrub typhus, an endemic disease in the Asia-Pacific region, is an important acute febrile illness in the tropics [42]. Development of an animal model that mimics the human histopathology and bacterial distribution is an important step toward understanding disease pathogenesis and immunity, as well as developing preclinical evaluation and interventions to prevent the infection and ameliorate its severity. Mouse models available to study scrub typhus have employed mostly the i.p. inoculation route, which results in infection that does not resemble the human disease clinically, target organs and cells, and histopathology. Our group recently developed an i.v. inoculation model of OtK infection, resulting in hematogenously disseminated endothelial infection mimicking human disease [31]. This new model has permitted us to examine how endothelial stress and dysfunction [31,32], or alarmin molecules such as IL-33 [43], contribute to oriential pathogenesis.

Since natural infections are initiated via mite feeding on the dermis of the skin, we sought to develop an i.d. inoculation model of scrub typhus using OtK. In this report, we have shown that following a 10-day incubation period, i.d.-inoculated mice developed a systemic infection with body temperature changes, weight loss, and bacterial dissemination via the blood to other major organs (Figs 1 and 2). While the clinical features of OtK-infected mice closely mimic the course of infection observed in human scrub typhus [44,45], the duration of fever and hematological abnormality appeared to be much shorter or milder in these mice as compared to human cases [46]. Interestingly, these mice developed a persistent infection with histologic lesions and infectious bacteria up to 84 dpi. To the best of our knowledge, this is the first report of a murine i.d. model for acute and persistent infection, which will be of great value for future immunologically or vaccine-based studies.

Our bacteriologic and histopathological analyses have revealed several important features of the i.d. infection model of *O*. *tsutsugamushi* (Figs 2–5). First, this inoculation route led to the establishment of infection with similar target cell tropism as scrub typhus, namely endothelial cells lining the microvasculature and macrophages, as in our previous report of the i.v. inoculation route and our study of human scrub typhus cases [2,31]. Secondly, while the lungs were the major target organ for the infection, containing 10-fold more *Orientia* 47-kDa gene copies than the liver, spleen, and brain samples, we observed considerable differences in lesion distribution and magnitude of inflammatory infiltrates. Bacteremia was sustained through 15 dpi, after the peak of parenchymal bacteria at 13 dpi, leading us to hypothesize that the delayed peak observed in the blood may be due to the release of *Orientia* from infected tissues. This is suggested by the observation that by 15 dpi parenchymal tissue bacterial loads decreased, which coincided with a bacterial load increase in the circulating blood. However, given that bacteremia kinetics mirror parenchymal tissue bacterial loads at the acute stage of the disease, further analysis with perfused organs would be informative. Thirdly, the histologic pattern of lung lesions was that of acute interstitial pneumonitis, which resembles the histopathology in human scrub typhus patients and in experimentally infected, non-human primates [4,22,23,47]. Histological lesions in the mouse lungs had not resolved completely even at 77–84 dpi, by which time most cytokines and chemokines had returned to their basal levels. Finally, the liver inflammatory index is a reliable scoring system for comparative studies, especially during the acute stages of infection. Detection of *Orientia* DNA in the liver, spleen and brain at 63–70 dpi poses the question as to the cell type(s) persistently infected in these organs and the long-term impact of persistent infection. Such information will be important to understand the potential complications of persistent *Orientia* infection in humans [16,48].

We detected a panel of Th1- and Th2-promoting, pro-inflammatory cytokines and chemokines in serum samples and lung homogenates (Figs 6 and 7). Classical type 1 cytokines and chemokines (e.g., IL-2, IL-12, IFN-γ, TNF-α, MCP-1/CCL2, MIP-1α/CCL3, and RANTES/CCL5) were predominantly induced around the peak of bacterial infection, and then decreased as bacterial replication was controlled at 28 dpi. Type 1 immune responses regulate migration of neutrophils, monocytes/macrophages, and T cells from the bloodstream across the vascular endothelium, as well as their effector function for bacterial control. These cytokine/chemokine patterns resemble the observations in infected humans [49,50,51] and in i.d.-inoculated non-human primates [25].

In this i.d. model, three immune modulatory cytokines were of particular interest to us. First, IL-10 was one of the earliest cytokines, detected as early as 9 dpi in the blood, and one of the few cytokines with sustained and significant production for 8 days. While IL-10 may contribute to minimizing host tissue damage, it will be important to further examine whether IL-10 also contributed to bacterial persistence in multiple organs in our model. Additional studies with mice deficient in IL-10 or its receptor will provide new insight into the roles of IL-10 and its relevance to *Orientia* persistency. Secondly, IL-13 was detected in both blood and lungs, implying the development of Th1/Th2-balanced immune responses in the i.d.-inoculated mice. This finding is particularly exciting because IL-13 and IL-4 proteins or transcripts were undetectable, or were lower than the sham controls, in our previous studies with i.v.-inoculated mice [32]. Given that only two reports mentioned low IL-4 levels in serum samples of scrub typhus patients [49,50], our data presented here provide new insights into Th2 cytokines in inflamed tissues. Finally, the marked reduction of IL-9 in the lung at 9 dpi, but elevation of IL-9 in the blood at 15–17 dpi, is intriguing. IL-9 is a pleiotropic cytokine that has documented effects on lymphocytes, mast cells, and resident lung cells. IL-9 is mostly produced by a special subset of CD4^+^ T lymphocytes, known as Th9 cells, that can modulate host immune responses by producing IL-9 and IL-10 [52]. In T cells, the most efficient priming of IL-9 production occurs in response to a combination of TGF-β and IL-4 [52]. Other IL-9-promoting factors include IL-2, IL-1β, IL-6, and IL-10 (which were detected in the blood and lung samples), type 1 IFN, and IL-21. Although IL-9 is involved in inflammatory responses due to allergy or classical Th2 responses [53,54], its role in bacterial infections remains unclear. Regardless of the source or the role of IL-9, our data indicate that i.d. inoculation triggers Th1/Th2-balanced immune responses in the early stage of *O*. *tsutsugamushi* infection.

In summary, we have presented the first report of an inbred murine i.d. inoculation model that leads to systemic *O*. *tsutsugamushi* infection. The clinical signs during the acute stage as well as the histopathologic and immunologic changes resemble most features of human scrub typhus. The significance of this study is the establishment of a model employing the natural route of infection and the occurrence of bacterial persistence. A chronic or persistent infection model for *O*. *tsutsugamushi* infection on the B6 background will be of great value for mechanistic studies for immune regulation because of the availability of knockout mouse strains on this genetic background. Using this model, we anticipate that future research efforts may be directed at examining early events in infected skin and skin-draining lymph nodes as this model mimics transmission by mite bite [34,35]. The focus of future studies by our laboratory utilizing this model includes examining homologous and/or heterologous protection via the cutaneous challenge and exploring vaccines to prevent acute and persistent infections. This study offers a greatly improved i.d. murine model for *Orientia* infection and will allow detailed studies of immune regulation and infection control.

## Supporting Information

S1 Fig Seroconversion following infection with *O*. *tsutsugamushi*. Reciprocal IgG endpoint titers of serum from sham control mice (n = 3) and infected mice (n = 4) was measured by indirect IFA. The bars represent means for the given groups.
(TIFF)

S2 Fig Bacterial loads in the brain and kidneys following infection with *O*. *tsutsugamushi*. Quantification of the *Orientia* p47 gene in the brain and kidneys by qPCR. Data are presented as 47-kDa gene copies/10^5^ GAPDH for tissues.
(TIFF)

S3 Fig Erythrocyte and thrombocyte responses following infection with *O*. *tsutsugamushi*. Hematologic parameters of whole blood from sham control mice (n = 3) and infected mice (n = 4) were measured using a 950FS HemaVet apparatus. Data are presented with values for individual animals, plotted and mean and standard deviation for each time-point. Dotted lines represent mean values for the sham control mice. PLT, platelet count; RBC, red blood cell count; HGB, hemoglobin; HCT, hematocrit; MPV, mean platelet volume; and RDW, red cell distribution width.
(TIFF)

S4 Fig Histopathologic changes in mouse kidney and spleen after infection with *O*. *tsutsugamushi*. **A**) Section of the renal cortex collected at 77 dpi, showing a cluster of inflammatory cells in the interstitium (100X). **B**) A high-power image revealed a collection of macrophages and occasional apoptotic bodies (400X). **C**) Spleen section collected at 9 dpi, showing hyperplasia of periarteriolar lymphoid sheaths and expansion of the marginal zone (100X). **D**) A high-power view of the central area of the lymphoid follicle in ***C***, showing numerous activated lymphocytes, immunoblasts and scattered apoptotic bodies (400X).
(TIFF)
