# Supplementary figures and images for: An Intradermal Inoculation Mouse Model for Immunological Investigations of Acute Scrub Typhus and Persistent Infection
Source: PLoS Negl Trop Dis. 2016 Aug 1;10(8):e0004884. doi: 10.1371/journal.pntd.0004884 (PMC4968841; doi:10.1371/journal.pntd.0004884)

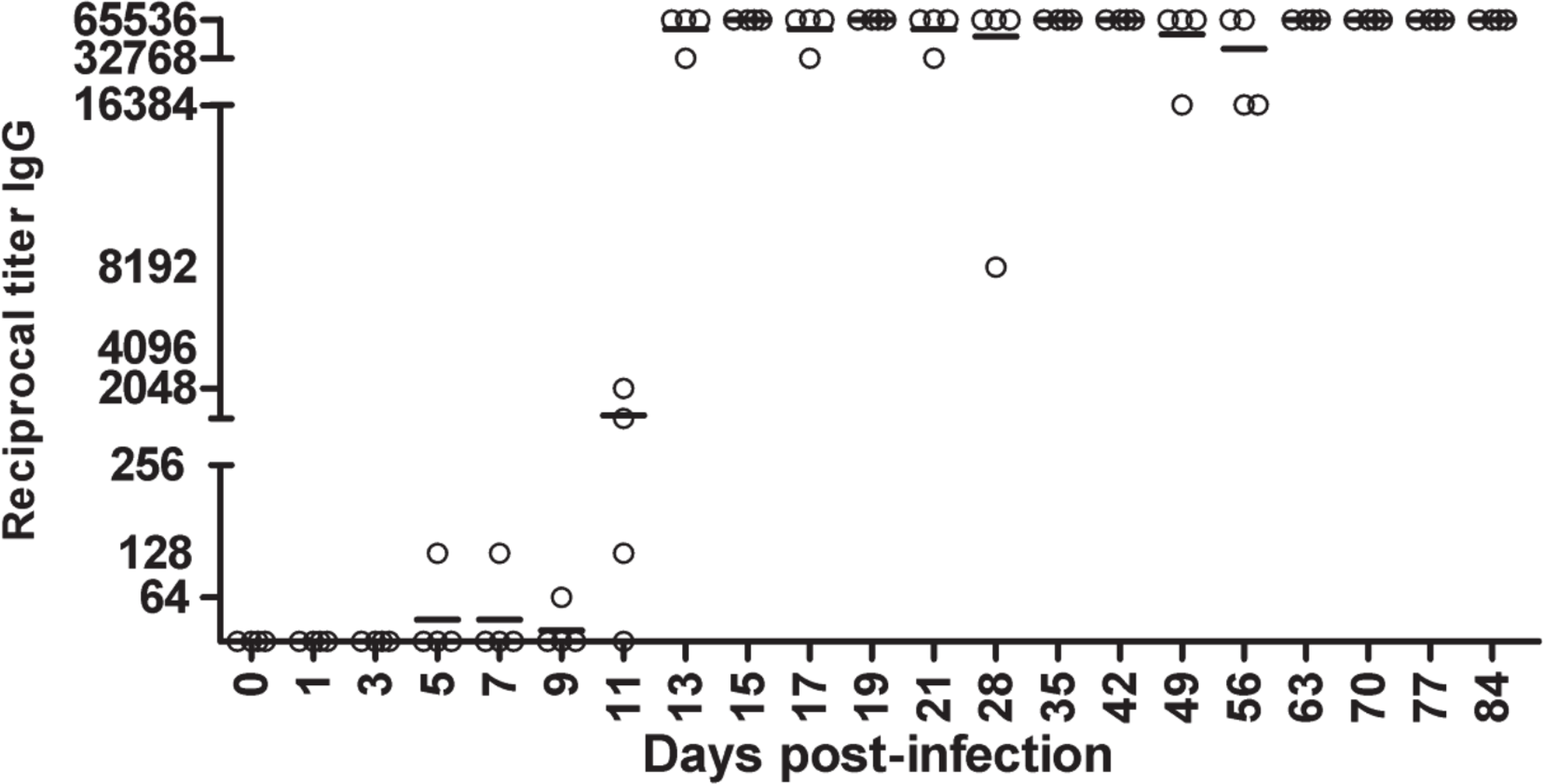

Supplement: S1 Fig — Reciprocal IgG endpoint titers of serum from sham control mice (n = 3) and infected mice (n = 4) was measured by indirect IFA. The bars represent means for the given groups. (TIFF) [file pntd.0004884.s001.tiff]

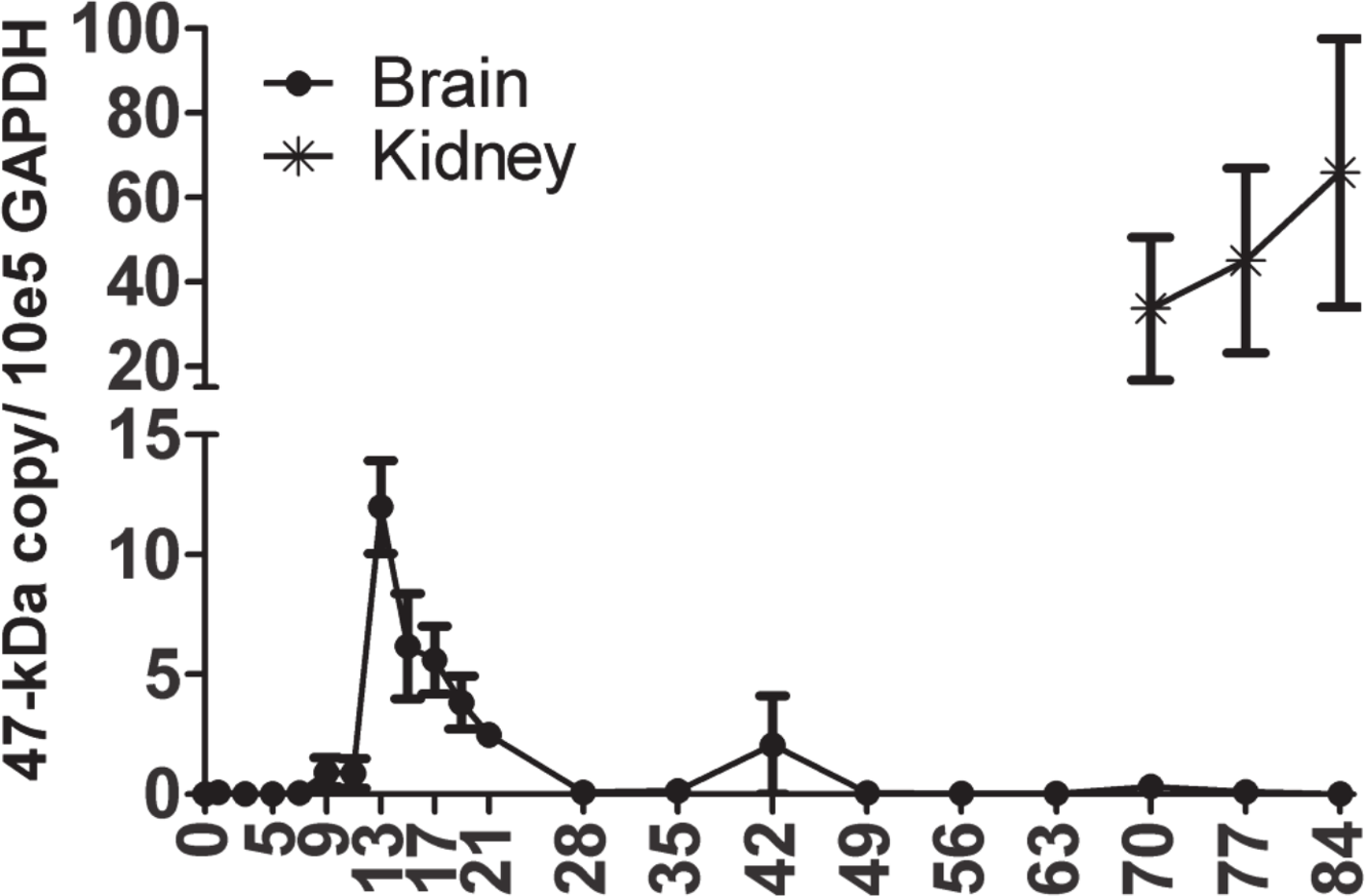

Supplement: S2 Fig — Quantification of the Orientia p47 gene in the brain and kidneys by qPCR. Data are presented as 47-kDa gene copies/105 GAPDH for tissues. (TIFF) [file pntd.0004884.s002.tiff]

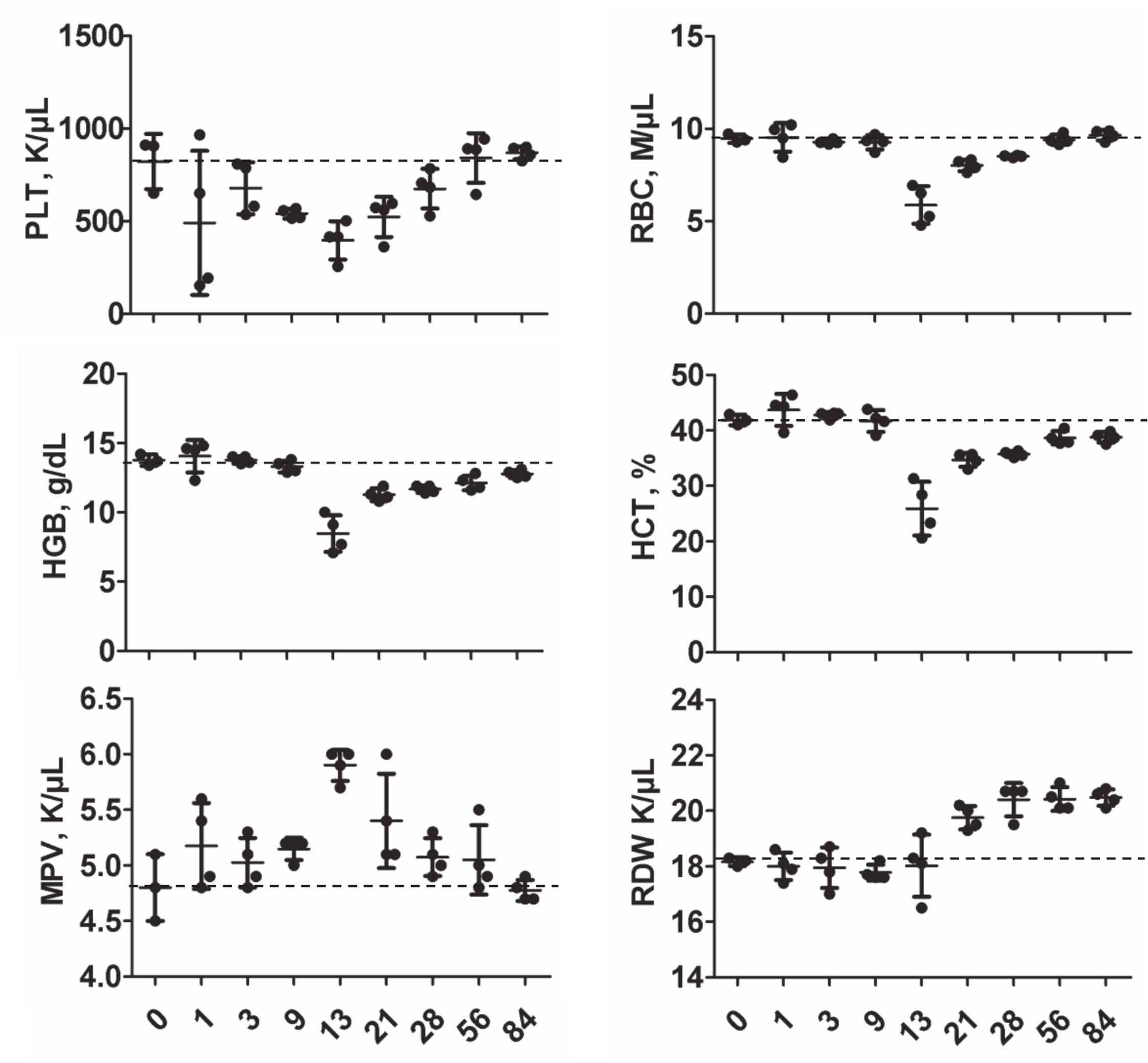

Supplement: S3 Fig — Hematologic parameters of whole blood from sham control mice (n = 3) and infected mice (n = 4) were measured using a 950FS HemaVet apparatus. Data are presented with values for individual animals, plotted and mean and standard deviation for each time-point. Dotted lines represent mean values for the sham control mice. PLT, platelet count; RBC, red blood cell count; HGB, hemoglobin; HCT, hematocrit; MPV, mean platelet volume; and RDW, red cell distribution width. (TIFF) [file pntd.0004884.s003.tiff]

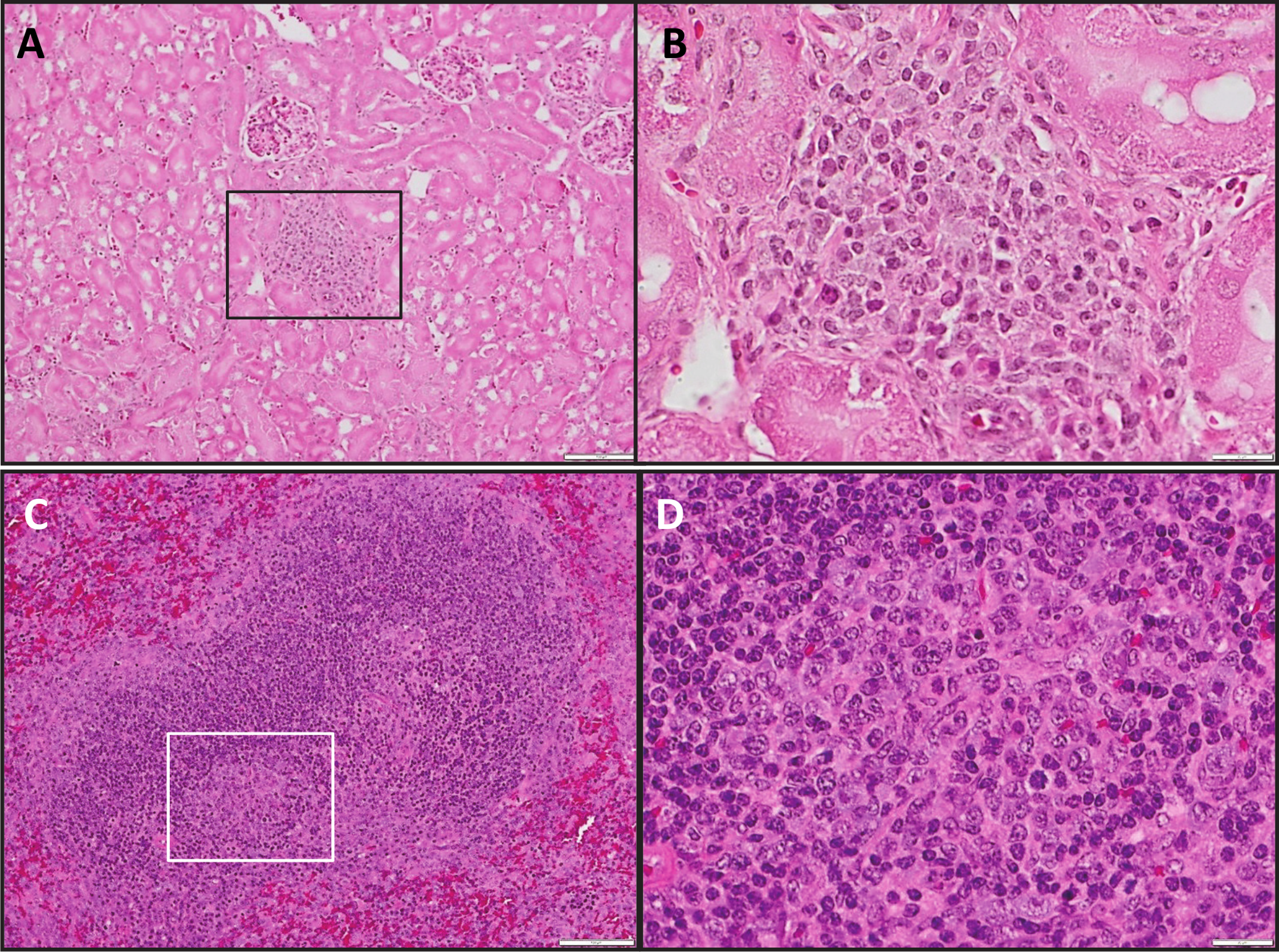

Supplement: S4 Fig — A) Section of the renal cortex collected at 77 dpi, showing a cluster of inflammatory cells in the interstitium (100X). B) A high-power image revealed a collection of macrophages and occasional apoptotic bodies (400X). C) Spleen section collected at 9 dpi, showing hyperplasia of periarteriolar lymphoid sheaths and expansion of the marginal zone (100X). D) A high-power view of the central area of the lymphoid follicle in C, showing numerous activated lymphocytes, immunoblasts and scattered apoptotic bodies (400X). (TIFF) [file pntd.0004884.s004.tiff]
